# Post digestion weed seed survival in cattle

**DOI:** 10.3389/fpls.2025.1483774

**Published:** 2025-03-18

**Authors:** Nebojša Nikolić, Selene Massaro, Franco Tagliapietra, Stefano Schiavon, Roberta Masin

**Affiliations:** Department of Agronomy, Food, Natural Resources, Animals and Environment (DAFNAE), University of Padova, Legnaro, Italy

**Keywords:** germinability, ruminant digestion, viability, weed seeds, *in vitro*

## Abstract

**Introduction:**

This research aimed to evaluate the impact of ruminant digestion on viability and germination pattern of the seeds of 9 weed species (*A. theophrasti*, *A. myosuroides*, *A. retroflexus*, *A. sterilis*, *C. album*, *D. stramonium*, *E. crus-galli*, *L. multiflorum* and *S. halepense*).

**Methods:**

One hundred seeds of each species were included in nylon bags and exposed to *in vitro* procedures simulating the rumen fermentation according to an experimental design that considered: 9 weed species, 3 incubation times in the artificial rumen (12, 24 and 48 h), 2 diets (lactating cows, and heifers), 4 replications, plus 4 additional replicas per species with seeds not subjected to the *in vitro* digestion as a control. This design was repeated two times (2 batches), involving a total of 504 replicas. Results were expressed in relative terms, using the data from the untreated seeds as a scaling factor. Data were analyzed, by species, with a model that considered diet and incubation time, and their interaction as fixed factors, and the batch as a random effect.

**Results and conclusions:**

Incubation time evidenced the greatest impact on seed germination and viability (6 species), where diet (4 species) and Incubation time x diet interaction (3 species) had lower impact. Compared to the control, *A. theophrasti* germination increased to 150% after 12 h but dropped to ~20% after 48 h under the lactating cows’ diet. Germination of *A. myosuroides* remained stable initially but fell to ~60% after 48 h, while *A. sterilis* showed consistently low germination, further declining with digestion. Germination of *C. album* rose to ~130% after 48 h, and the one of *E. crus-galli* to ~140%. For *D. stramonium*, germination decreased to ~20%, with heifers’ diet causing greater losses. No significant effects were noted for *L. multiflorum*, *S. halepense*, or *A. retroflexus*. Viability losses were significant for *A. theophrasti* and *A. myosuroides* under the lactating cows’ diet and for *D. stramonium* under the heifers’ diet. Possible variation causes were evidenced in the thickness and the fibrous content (NDF, ADF, ADL) of the seed coats, correlated with the rumen microbial activity.

## 1 Introduction

Animal husbandry is heavily dependent on plant production to provide feed for the animals and can be strongly influenced by farmland management and different agricultural practices (Makkar, 2018). In cattle production, different fodder types, such as hay, silage, grains, and other plant parts are used to ensure a balanced diet and maintain animal health. All of these fodders could contain a certain percentage of viable weed seeds which could be ingested by cattle (Blackshaw and Rode, 1991). Some of the main crops used in cattle rations are maize (*Zea mays* L.) and soybean (*Glycine max* [L.] Merr.), which could also potentially be infested by different weed species during their growth. Other than just reducing the yield of these important crops, some of the seeds of different weed species may contaminate the harvested crop seeds, eventually finding their way into the cattle fodder (Wilson et al., 2016; Anwar et al., 2021; Rubenstein et al., 2021). The presence of the weed seeds in these products is dependent on the harvest time and the storage of the obtained products. Therefore, the raw materials could contain up to 20% of whole and viable weed seeds (Blackshaw and Rode, 1991).

Previous research concluded that a significant number of weed species seeds could survive the passage through the animal digestive system and germinate afterwards (Salazar-Rivera et al., 2020; Lee et al., 2021). In some cases, the seeds that went through the animal digestive system became even more germinable, compared to the intact seeds, or were able to germinate faster (Katovich et al., 1993; Salazar-Rivera et al., 2020; Oveisi et al., 2021). Finally, after the processes inside the animals, the digested fodder, and with it, some of the still viable weed seeds, exits the animals as excrement. In various research conducted, a high proportion of viable weed seeds have been found in these excrements both in wild and farm animals (Cudney et al., 1992; Lee et al., 2021; Liu et al., 2020; Oveisi et al., 2021; Pleasant and Schlather, 1994; Rahimi et al., 2016; Salazar-Rivera et al., 2020). With farm animals, these excrements become part of manure and slurry, which are distributed in the fields as organic fertilizers (Du et al., 2020; Prado et al., 2022; Schröder, 2005; Schröder and Dilz, 1987). If some of the weed seeds are still viable after the passage through cattle digestive system, they could find their way into the fields as part of the manure or slurry, further increasing the weed infestation of the fields, which has been confirmed by different studies (Cudney et al., 1992; Katovich et al., 1993; Lowry, 1996; Materechera and Modiakgotla, 2006; Platis et al., 2023; Pleasant and Schlather, 1994). This comes as no surprise, considering that, once viable seeds have germinated, manure provides the nutrients that seedlings require for their development (Hogan and Phillips, 2011). Beyond its localized impact, the survival of weed species within the digestive systems of cattle could potentially facilitate the spread of these species on a larger scale. There is evidence that historically landscapes were shaped by grazing cattle at long distances, and also through cattle trade, bringing new plant species even across continents (Bruun and Fritzbøger, 2002; Fuller and Boivin, 2009). In addition, the potential for introduced species to become invasive due to their absence in the new environment increases the risk of introducing resistant weed species to novel areas. This increased risk poses a significant threat to both agricultural production and agroecosystems (Andrews, 1995; Bruun and Fritzbøger, 2002; De-Clerck-Floate, 1997; Fuller and Boivin, 2009; Hogan and Phillips, 2011; Malo and Suárez, 1995; Rahimi et al., 2016).

Given the various issues and adverse effects associated with the presence of weed seeds in cattle diets, it is crucial to gain a deeper understanding of the fate of seeds from key weed species after they pass through the cattle digestive system. This serves as the starting point for identifying livestock and manure management strategies to effectively reduce weed seed viability, defined as both germinable and viable but dormant seeds, and their spread. Considering the major crops grown in the region, used also as animal fodder, more precisely maize (*Zea mays*) and soybean (*Glycine max*) it was decided to focus on some of the agronomically most important weed species of these crops in north-eastern Italy. Therefore, this study aimed to evaluate the degradation and the germination of nine weed species seeds (*Abutilon theophrasti* Medik.; *Alopecurus myosuroides* Huds.; *Amaranthus retroflexus* L.; *Avena sterilis* L.; *Chenopodium album* L.; *Datura stramonium* L.; *Echinochloa crus-galli* [L.] P. Beauv.; *Lolium multiflorum* Lam.; *Sorghum halepense* L.) after different times (12, 24, and 48 hours) of permanence in the rumen fluid with diets for lactating cows and heifers, and after the post-ruminal digestion with *in vitro* trials.

## 2 Materials and methods

### 2.1 Ethics statement

All the experimental procedures were carried out according to the Italian law on animal care and welfare. In order to obtain the rumen fluid for *in vitro* fermentations, all the practices were approved by the ethical committee at the University of Padova (OPBA, approval number 1312041/2022).

### 2.2 Experimental design

Mature seeds of nine weed species, *Abutilon theophrasti*, *Alopecurus myosuroides*, *Amaranthus retroflexus*, *Avena sterilis*, *Chenopodium album*, *Datura stramonium*, *Echinochloa crus-galli*, *Lolium multiflorum* and *Sorghum halepense*, were harvested by hand, during the season of 2021, at the experimental farm “Lucio Toniolo” of the University of Padova situated in Legnaro, Italy. Following harvest, the seeds were cleaned using a two-step process. Initially, they were manually processed by rubbing against a wooden board fitted with a rubber lining to prevent damage. This step helped grind and reduce the weight of various impurities. Subsequently, a seed blower was employed to separate the seeds from the remaining impurities by blowing away the lighter particles. After completing the cleaning process, the seeds were stored in a dry environment at 4°C before being subjected to the *in vitro* rumen fermentation. The experimental setup involved 4 replicates for each of 9 seed species × 3 incubation times × 2 diets. In addition, there were 4 additional replicates per species as untreated controls, i.e. not subjected to *in vitro* rumen fermentation (36 replicates). The data obtained from the control replicates were used to estimate the dormancy and viability of the weed species tested (**Supplementary Table S1**). This experimental plan was repeated two times (2 batches), for a total of 504 replicas, 56 replicas per each species. Each bag (replicate) comprised 100 seeds of the same species.

The seeds of each replicate, used for the *in vitro* simulation of rumen fermentation, were enclosed in woven nylon bags with a porosity of 50 ± 10 μm, manufactured by Ankom (Ankom Technology^®^, Macedon, NY, USA). The nylon bags, proven effective in previous analyses, permit the entrance of the rumen fluid microorganisms into the bag, while securely containing the studied material (Weakley et al., 1983; Vogel et al., 1999). To prevent potential degradation and bag opening that could lead to seed loss, the seeds were tightly secured within the bags using nylon thread after placement.

### 2.3 Rumen fluids sampling, diets, and medium preparation

Rumen fluid (RF), was sampled, according to consolidated procedures (Tagliapietra et al., 2012) using an esophageal probe by two fasting Simmental cows and by two Simmental heifers in the experimental farm “Lucio Toniolo” of the University of Padova (Legnaro, Italy). The collected RF (approximately 1-L per animal) was transported from the field to the laboratory, maintaining the temperature of 39°C in thermos. When the RF arrived in the laboratory, it was filtered with 4 layers of cheesecloth to remove the particles and then maintained under a flux of carbon dioxide (CO_2_) and at 39°C in a warm bath.

The degradative impact of rumen incubation was assessed for both lactating cow and heifer diets. Samples representative of the two diets were prepared by mixing the single ingredients (dried and milled at 1 mm screen) in the proportions reported in **Table 1**. The medium solution was prepared according to Menke and Steingass (1988). The medium solution was kept at 39°C under a flux of CO_2_ until the arrival of the RF in the laboratory.

**Table 1:** Ingredient composition (% DM) of the two diets used in the experiment.

Ingredients	Lactating cows	Heifers
Corn silage	20.1	–
Grass silage	3.8	15.5
Sorghum silage	31.5	6.2
Alfalfa hay	6.0	–
Ryegrass hay	–	47.0
Corn Gluten feed	6.1	16.0
Energy mixture (corn-barley)	11.7	–
Protein mixture (soy, sunflower)	16.1	–
Soybean meal	–	13.5
Vit-Mineral mix and wheat/flax germ	1.3	–
Vitamin supplement	**-**	1.8
Molasses	3.4	–
Total	100	100

### 2.4 *In vitro* fermentation

The *in vitro* fermentations were performed using the Daisy^II^ incubator (Ankom Technology ^®^, Macedon, NY, USA), equipped with four vessels. The system is designed to maintain stable anaerobic and temperature conditions and it provides measurements of fiber degradability closely correlated to the fiber degradability measured *in situ* (Spanghero et al., 2003).

For our experiment, we used only two vessels, one designated to simulate the rumen fermentation of a lactating cow diet and the other one that of the heifers. Before the incubation process, the samples of RF collected from the two lactating cows were pooled, as well as the RF samples from the two heifers, resulting in two distinct RFs that were poured together with 12.5 g of dry matter (DM) of each diet in the vessel.

The anaerobic and temperature conditions in the vessels were assured by purging CO_2_ and preheating all the materials and fluids at 39°C before incubation. The vessels were filled with 800 mL of the respective RF and 1600 mL of the medium solution, according to the proportions indicated by Menke and Steingass (1988). In each batch, 108 bags (4 replicates × 9 species × 3 incubation times) containing the weed seeds were incubated in each vessel for 12, 24, or 48 h. At each incubation time, a third of the bags were removed from the fermentation vessels. The three incubation times were used to evidence differences in the loss of germinability and viability of the fodders weed seeds up to 48 h, which is the conventional duration to assess the ruminal degradability of fodders (Van Soest and Wine, 1967).

### 2.5 Post ruminal digestion

Huhtanen and Vanhatalo (1997) indicated that post ruminal digestion contributes less than 5% to the degradation of the fibrous constituents, and consequently we can assume that it has a comparable impact on seed scarification. To simulate post-ruminal digestion in cattle, the methodology proposed by Boisen and Fernández (1997) was used. The 54 bags removed from the Daisy incubator at each incubation time were placed in a 5-litre plastic beaker and exposed to an *in vitro* simulated gastric environment. The beaker was filled with a solution consisting of two liters of a 0.1M phosphate buffer plus approximately 10 ml of 0.2 M HCl to adjust the solution pH to 2.0. One ml of a fresh solution containing 25 mg of pepsin (porcine pepsin, 2000 FIP-U/g, Merck, Darmstadt, Germany) was added to this solution. The nylon bags extracted from the Daisy incubator were immersed in this solution for 4 hours.

To simulate digestion in the small intestine, after the fourth hour, the pH of the solution containing the nylon bags was raised to 6.8 by adding 10 ml of a 0.2 M phosphate buffer at pH 6.8 and approximately 5 ml of 0.6 M NaOH. One ml of a fresh solution containing 100 mg of pancreatin (porcine pancreatin, Merck, Darmstadt, Germany) was then added to the solution. The bags with the seeds were exposed to this solution for 7.5 hours.

To simulate large intestine digestion, 0.5 kg of fresh feces from the experimental lactating cows and heifers were collected and separately mixed with 1 liter of a buffer solution prepared as described by Menke and Steingass (1988). At the end of the simulated small intestine digestion, the nylon bags were immersed in this mixture for 3.5 hours. After that, the bags were removed from the mixture, washed with warm water, and prepared for the germination test.

### 2.6 Germination tests

In each batch, after the rumen and post-rumen digestion, the seeds were sown onto filter paper saturated with 2 mL of distilled water in individual Petri dishes. Each bag resulting from the previous treatments was assigned to a Petri dish. In addition, 36 control Petri dishes containing the weed seeds of the 9 species (four for each species) not subjected to the *in vitro* digestion procedures were included as control. After sowing, the Petri dishes were sealed with parafilm and placed inside germination chambers. Two alternating temperature regimes were employed based on the optimal germination temperatures for each species, maintaining a 12-hour light/12-hour dark photoperiod. Specifically, temperatures of 15°C/25°C were maintained for *A. myosuroides*, *A. sterilis*, *C. album*, and *L. multiflorum*, while 18°C/30°C were applied for *A. theophrasti*, *A. retroflexus*, *D. stramonium*, *E. crus-galli*, and *S. halepense*. The selection of these specific temperature ranges is based on the germination periods of the species studied. The first four species are generally classified as spring species, while the latter four are categorized as summer species. Consequently, the chosen temperatures were intended to replicate the optimal conditions for germination and growth of these species, as demonstrated in studies by Nikolić et al. (2022) and Song et al. (2024). Germination progress was monitored every 2-3 days, counting and removing germinated seeds and additional water was added if necessary. Germination trials were considered complete when all seeds germinated or after 10 d without germination (Baskin and Baskin, 2014).

Upon conclusion of germination, non-germinated seeds underwent a tetrazolium test (Borza et al., 2007) to determine their viability, distinguishing between dormant viable seeds and those that had lost viability. Statistical analyses were performed on both the germinated seeds and the viable seeds (germinated + viable after the tetrazolium test) to comprehensively assess the impact of passage through the cattle digestive system on the survival and germination of weed species seeds. To optimize the dataset and highlight the possible effects of different treatments on the germination and viability of different species, the number of germinated and viable seeds for each species under treatment was expressed as percentage on the respective untreated control.

### 2.7 Chemical analysis

Given the extensive literature linking seed viability and germination to the chemical composition of seeds, these analyses were conducted to complement the findings of this study. By assessing the chemical composition of the seeds, we aimed to contextualize the observed results and compare them with existing research, for a more comprehensive understanding of the factors influencing seed survival and germination.

For this, samples (5 g) of all the weed seeds used in the two years were collected and grounded at 1 mm (Retsch ZM 200, Retsch GmbH, Germany) to be analyzed.

The sample DM was determined using an oven at 101-103°C overnight (AOAC, 2016; method 978.01). Ash content was obtained after placing the samples in a muffle at 550°C for 4 hours and weighted after cooling in a desiccator (AOAC, 2016; method 942.05). Crude protein (CP) content was measured according to the Kjeldahal method (AOAC, 2016; method 978.04) using a Kjeltec™ 8400 apparatus (Foss Electric A/S, Hillerød, Denmark). Ether extract (EE) was obtained according to the AOAC guidelines (AOAC, 2016; method 2003.05).

Fiber fractions were determined using the Van Soest methodology (Van Soest et al., 1991). Neutral detergent fiber (aNDF) and acid detergent fiber (ADF) were performed sequentially using the Ankom fiber analyzer (Ankom, Rochester, NY, USA), and following the Ankom procedures (Ankom Technology, 2023). Neutral detergent fiber was determined with heat-stable α-amylase and without sulfite (aNDF) (AOAC, 2000; method 2002.04). Acid detergent lignin (ADL) was obtained after ADF analysis using the analytical method proposed by Ankom (Ankom Technology, 2022). After all the determinations, the hemicellulose and the cellulose content of each sample were calculated:


Hemicellulose (%DM)=aNDF–ADF

Cellulose (% DM)=ADF–(ADL+AIA)

where AIA indicates the acid insoluble ash.

The starch content was also determined using an enzymatic method via HPLC (High Pressure Liquid Chromatography), developing from the method proposed by AOAC (2016; method 996.11) and (Hall, 2000). All the samples were analyzed in duplicate. One gram of samples was weighed and dissolved with 50 mL of potassium hydroxide 0.5M for 1 hour at 60°C, and, after that, the pH was raised to 4.5. One of the two duplicates of each sample was incubated without enzyme, and the second was inoculated with amino-glucosidase and incubated (8 hours at 40°C). At the end of the 8 hours, the samples were filtered and injected in HPLC. The sample without inoculation provided the amount of free glucose. The sample with the inoculum provided the amount of total glucose. Subtracting the free glucose from the total glucose we obtained the starch.

The rumen fluids and feces collected from dairy cows and heifers, used as microbial inoculums in *in vitro* fermentations, were analyzed for pH in triplicate. For each sample, 4 mL of rumen fluid and feces (diluted and homogenized 1:5 with distilled water) were collected in triplicates and stored at -30°C after the addition of 1 mL of metaphosphoric acid solution (25% w/v) to stabilize them prior to analysis. Ammonia nitrogen (N) concentrations were quantified using the ammonia rapid assay kit (K-AMIAR 02/20; Megazyme, Bray, Ireland). Volatile fatty acid (VFA) concentrations were determined via high-performance liquid chromatography (HPLC) using a Jasco system equipped with a PU-2080 pump (Jasco, Tokyo, Japan), a model RI-2031 refractive index detector, a model AS-2055 autosampler, and a model CO-2060 column oven. The chromatographic separation was carried out using an Aminex HPX 87H column (300 mm × 7.8 mm; Bio-Rad Laboratories, Hercules, CA, USA). Data acquisition and analysis were performed using ChromNAV software (Version 2.0, Jasco).

### 2.8 Rumen microbial activity

The cellulolytic microbial activity, which was estimated as a proxy for seed degradation, was quantified as described by Nikolić et al. (2024) by measuring the resistance to breakage of cotton threads incubated in the Daisy System together with the weed seeds. The cotton threads (n.16, Cucirini Tre Stelle, Caleppio di Settala, Milan, Italy) were cut to a length of 10 cm and placed into the Ankom nylon bags (5 × 5 cm; porosity of 50 ± 10 μm, Ankom Technology^®^, Macedon, NY, USA). The bags, each containing 6 cotton threads, were securely stitched to prevent thread loss and incubated for 12, 24 or 48 h together with the weed seeds. In each batch, 24 bags were incubated (2 diets × 3 incubation times × 4 replications). After exposure to the rumen microbial activity, the bags were opened, and the threads were retrieved and air-dried. Once dry, their resistance to breakage was measured using a digital dynamometer (IMADA ZP, ELIS Electronic Instruments and Systems, Rome, Italy). For the measurement, each thread was fixed to a keyring by pinching one end and coiling the thread several times around the ring. The dynamometer was set to the “peak” function mode, signifying the maximum load endured by the thread before breaking. This peak force was then determined by pulling the ends of the thread until they broke. In addition to the treated threads, non-treated cotton and silk threads were measured as standards to ascertain the increased fragility of the treated threads. The data obtained from the measurements of the treated threads were then compared to the average values of the non-treated thread standards to estimate the degree of microbial cellulolytic activity.

### 2.9 Statistical analysis

Statistical analyses were performed within the R environment (RStudio Team, 2023). Data of viability and germination of the seeds, separately for each species, were analyzed with an ANOVA model that included as fixed factors the incubation time (I; 2 d.f.), diet (D; 1 d.f.), batch (1 d.f.) and the incubation time × batch interaction (2 d.f.), after a check for homoscedasticity completed with the Leven test. The experimental unit was the single bag, containing 100 seeds. Data from the experiments (batches) were pooled as ANOVA showed no significant difference in treatment x experiment interactions. *Post hoc* analysis for significant mean differences was conducted using the Tukey *post-hoc* test for multiple comparisons, while the correlation analysis was conducted using the cor.test function of the R stats package.

The Fertimetro^®^ data, analyzed with a similar model that considered as fixed factors the diet, the incubation time and their interaction, were already published in Nikolić et al. (2024). The least square means resulting from this analysis were used to evaluate the correlation between the seed viability and the threads degradation traits, representing the rumen microbial activity.

## 3 Results

### 3.1 Chemical composition of weed seeds

The chemical composition of the inoculums used to simulate *in vitro* the fermentation occurring in the rumen and the large intestine of dairy cows and heifers is reported in **Table 2**. Feces contain higher amounts of ammonia nitrogen compared to ruminal fluid but lower concentrations of volatile fatty acids (VFA). Lactating cows, in comparison to heifers, exhibit significantly higher VFA concentrations, lower pH values and comparable N-NH3 concentrations in both ruminal fluid and feces.

**Table 2:** Rumen fluid and faeces content of ammonia nitrogen (N-NH3, mmol/L) and volatile fatty acid (VFA, g/L) and pH collected from lactating cows and heifers and used as inoculum for *in vitro* incubations. Each value is the mean of 3 replicate samples.

	Rumen fluid				Faeces			
	Lactating cows		Heifers		Lactating cows		Heifers	
	Mean	SD	Mean	SD	Mean	SD	Mean	SD
N-NH3, mmol/L	8.00	0.05	8.12	0.02	11.66	0.22	13.03	0.15
Total VFA^1^, g/L	8.64	0.21	6.78	0.38	5.26	0.01	3.22	0.28
Acetic acid, %	52.2	5.20	50.4	6.20	66.7	4.60	52.2	3.60
Propionic acid, %	20.2	4.10	21.9	3.50	17.6	2.30	15.4	1.21
Iso-butyric acid, %	0.9	0.05	3.7	0.35	1.1	0.06	2.8	0.36
Butyric acid, %	19.6	2.12	17.0	1.25	11.1	1.01	11.9	0.89
Iso-valeric Acid, %	2.5	0.07	2.3	0.36	0.4	0.05	2.0	0.05
Valeric acid, %	2.2	0.08	2.5	0.27	1.7	0.32	2.1	0.12
Caproic acid, %	2.1	1.00	2.0	1.69	1.2	0.24	6.3	2.60
pH	5.8	0.23	7.2	0.25	6.2	0.35	7.3	0.12

^1^ Volatile fatty acids.

The chemical composition of the seeds is detailed in **Table 3**. Acid Detergent Fiber (ADF) varied from a minimum of 10.31% in *L. multiflorum* to a maximum of 39.12% in *D. stramonium*, and was high in *A. theophrasti*, *C. album* and *S. halepense*. Lignin content peaked 24.14% in the seeds of *C. album*, while the content of hemicellulose and cellulose in these species was low. Moreover, the protein content is elevated in *A. theophrasti* (CP 21.19%). The seed composition of each species will be discussed afterwards in the paper in relation to the viability and germination response after the transit through the digestive tract of cattle.

**Table 3:** Chemical composition of the weed species seeds (% DM).

Constituent^1^	Species^2^								
	ABUTH	ALOMY	AMARE	AVEST	CHEAL	DATST	ECHCG	LOLMU	SORHA
DM % as fed	92.36	92.11	89.55	91.50	89.95	93.82	91.64	90.81	91.25
Ash	4.87	10.62	3.19	3.07	4.05	2.84	3.18	3.91	5.09
CP	21.19	11.34	16.45	15.64	15.14	15.98	10.02	16.52	10.91
EE	17.01	10.51	7.37	8.11	7.53	24.18	6.05	1.75	3.13
Starch	0.23	25.10	50.26	41.49	34.48	0.26	40.95	45.29	27.27
aNDF	45.74	35.74	22.69	27.03	33.37	49.42	34.48	27.68	47.82
ADF	29.07	19.86	16.95	11.82	29.16	39.12	20.42	10.31	28.08
AIA	0.17	2.36	0.40	0.52	0.42	0.28	0.35	0.17	1.19
Hemicellulose	16.67	15.88	5.73	15.20	4.21	10.31	14.06	17.37	19.74
Cellulose	18.27	12.47	4.17	8.99	4.59	16.47	14.40	7.73	15.98
Lignin	10.63	5.03	12.38	2.32	24.14	22.36	5.68	2.41	10.91

^1^ DM, Dry matter; CP, Crude protein; EE, Ether Extract; CF, Crude fiber; aNDF, Neutral detergent fiber with heat-stable alpha-amylase and without sulphite; ADF, Acid detergent fiber; AIA, Acid insoluble ash.

^2^ ABUTH, *Abutilon theophrasti* Medik.; ALOMY, *Alopecurus myosuroides* Huds.; AMARE, *Amaranthus retroflexus* L.; AVEST, *Avena sterilis* L.; CHEAL, *Chenopodium album* L.; DATST, *Datura stramonium* L.; ECHCG, *Echinochloa crus-galli* [L.] P. Beauv.; LOLMU, *Lolium multiflorum* Lam.; SORHA, *Sorghum halepense* L.

### 3.2 Seed germination and viability

**Figure 1:**
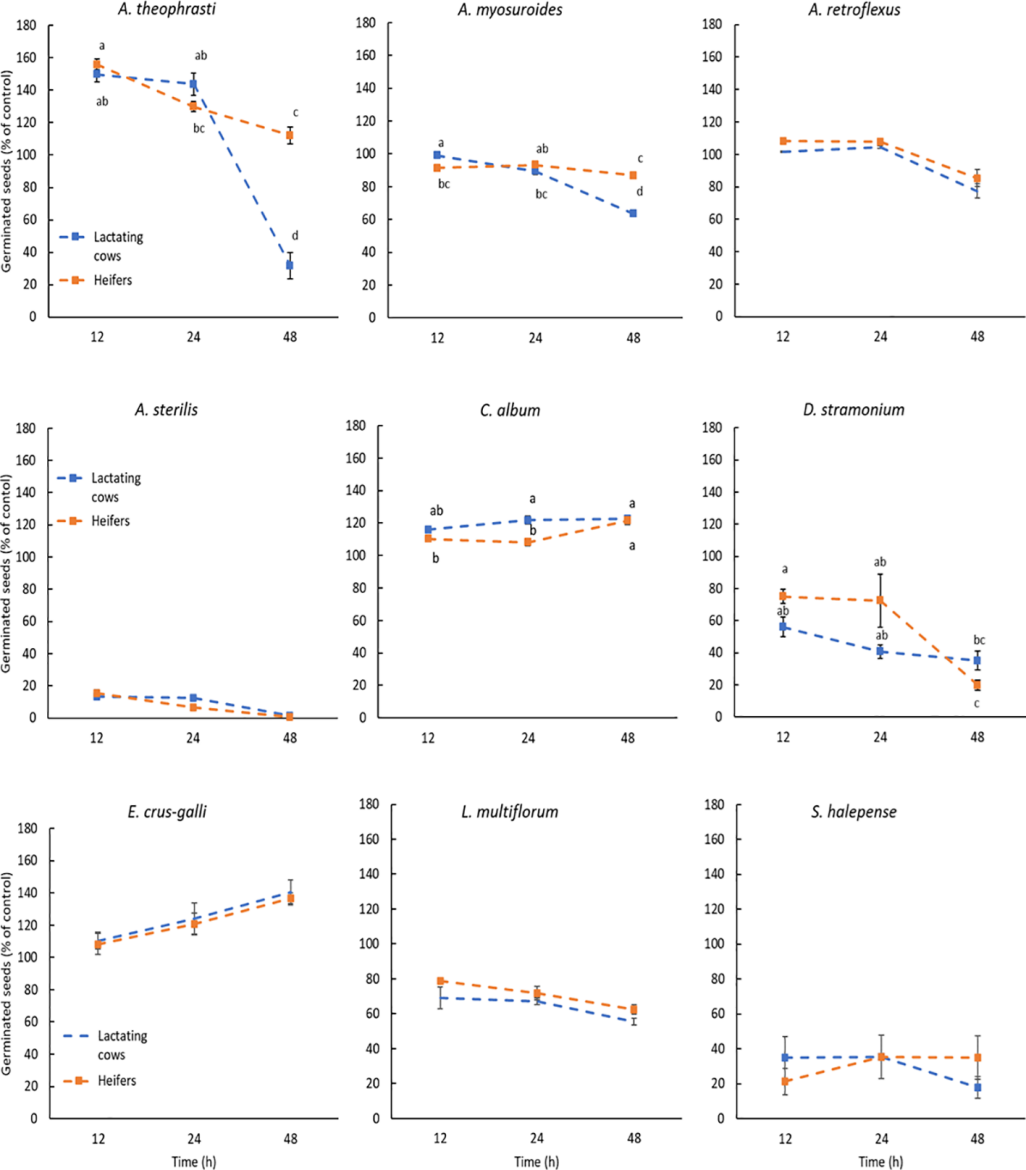
Germination of the species studied after the passage through cattle digestive system. The results are expressed as percentage of control. Different letters are reported when the Diet x incubation time interaction was significant (P-values< 0.05). Statistical difference due to the Diet and the incubation time are provided in **Table 3**. Error bars represent the standard error.

**Figure 2:**
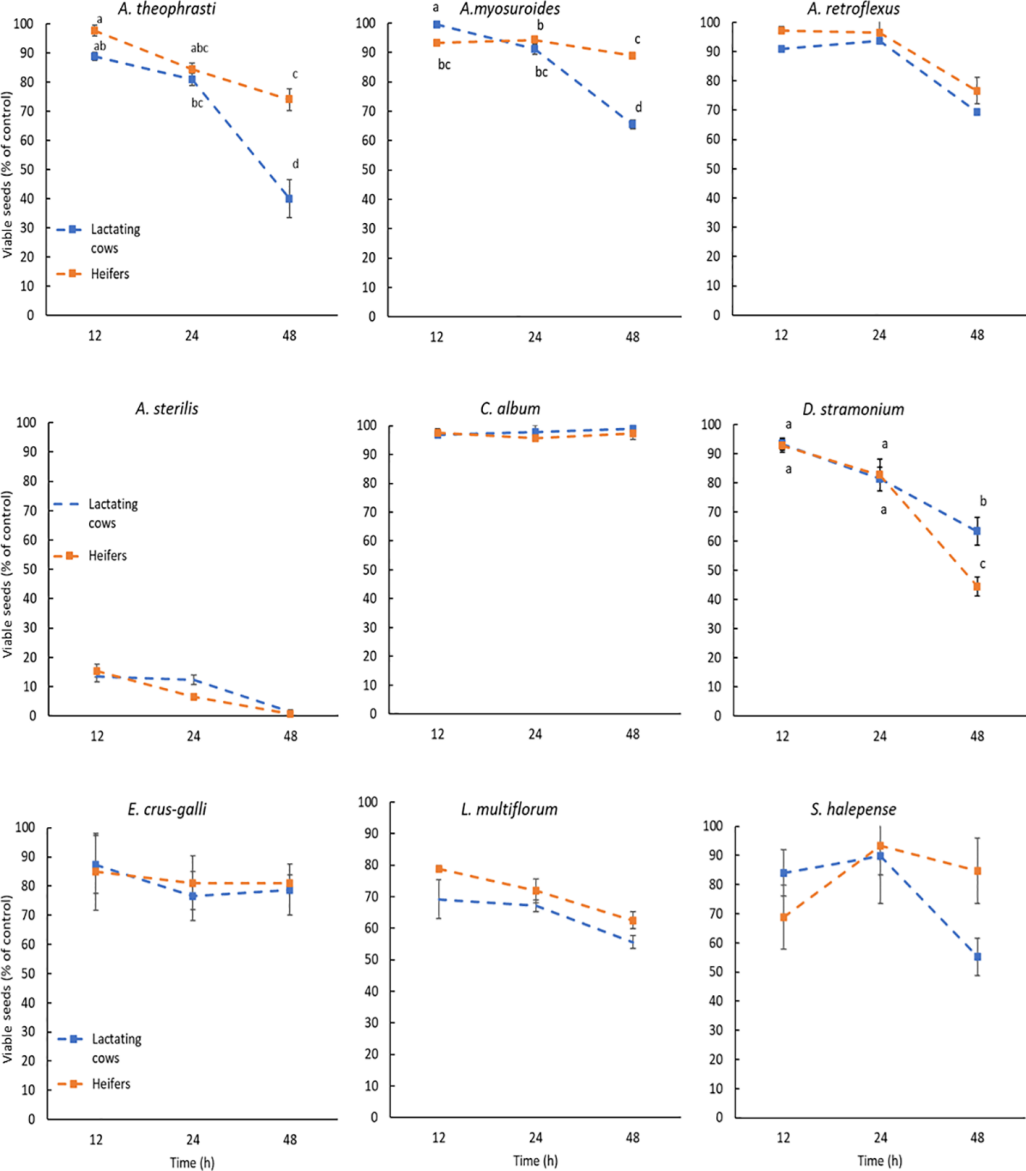
Viability of the species studied after the passage through cattle digestive system. The results are expressed as percentage of control. Different letters are reported when the diet x incubation time interaction was significant (P-values< 0.05). Statistical difference due to the diet and the incubation time are provided in **Table 3**. Error bars represent the standard error.

Seed germination and viability after the digestive processes are illustrated in the **Figures 1**, **2**. Different letters indicate a significant difference and are placed only where the interaction I x D was significant (**Tables 4**, **5**). Compared to control, the germination percentage of *A. theophrasti* seeds was increased (150%) for short exposure to the simulated rumen microbial activity, but it decreased for prolonged incubations (**Figure 1**). Notably, the diet for lactating cows had a more pronounced negative effect on the germinability of *A. theophrasti* seeds compared to the diet for heifers during extended seed residence in the rumen. Consistently with the findings on germination, the viability of *A. theophrasti* seeds (**Figure 2**), was significantly influenced by the incubation time (P< 0.001) and diet (P< 0.001), along with their interaction (P< 0.001). The viability of *A. theophrasti* seeds remained high after 24 hours in the rumen, while it decreased after 48 hours of rumen residence with both diets.

**Table 4:** Influence of different factors on seed germination of the studied species (P-values).

Species^1^	Factors		
	Incubation time (I)	Diet (D)	I × D
ABUTH	< 0.001	< 0.001	< 0.001
ALOMY	< 0.001	< 0.001	< 0.001
AMARE	< 0.001	0.023	0.738
AVEST	< 0.001	0.128	0.053
CHEAL	< 0.001	< 0.001	0.014
DATST	< 0.001	0.792	0.012
ECHCG	0.001	0.622	0.995
LOLMU	0.004	0.007	0.646
SORHA	0.173	0.968	0.532

^1^ ABUTH, *Abutilon theophrasti* Medik.; ALOMY, *Alopecurus myosuroides* Huds. AMARE, *Amaranthus retroflexus* L.; AVEST, *Avena sterilis* L.; CHEAL, *Chenopodium album* L.; DATST, *Datura stramonium* L.; ECHCG, *Echinochloa crus-galli* [L.] P. Beauv.; LOLMU, *Lolium multiflorum* Lam.; SORHA, *Sorghum halepense* L.

**Table 5:** Influence of different factors on seed vitality of the studied weed species (P-values).

Species^1^	Factors		
	Incubation time (I)	Diet (D)	I × D
ABUTH	< 0.001	< 0.001	< 0.001
ALOMY	< 0.001	< 0.001	< 0.001
AMARE	< 0.001	0.021	0.731
AVEST	< 0.001	0.128	0.053
CHEAL	0.316	0.188	0.228
DATST	< 0.001	0.074	0.031
ECHCG	0.086	0.630	0.599
LOLMU	0.004	0.007	0.646
SORHA	0.123	0.493	0.122

^1^ ABUTH, *Abutilon theophrasti* Medik.; ALOMY, *Alopecurus myosuroides* Huds. AMARE, *Amaranthus retroflexus* L.; AVEST, *Avena sterilis* L.; CHEAL, *Chenopodium album* L.; DATST, *Datura stramonium* L.; ECHCG, *Echinochloa crus-galli* [L.] P. Beauv.; LOLMU, *Lolium multiflorum* Lam.; SORHA, *Sorghum halepense* L.

Similar to the findings for *A. theophrasti*, both incubation time and diet, and their interaction, had a significant impact on the germination and the viability of *A. myosuroides* seeds. The germinability of *A. myosuroides* remained relatively stable for short and medium periods of residence in buffered rumen fluid, with a significant decrease after extended permanence. Notably, a more pronounced reduction in germination was evident for lactating cows compared to heifers. Additionally, it is noteworthy that there was little or no induction to germination after the passage through the simulated cattle digestive system for this species. The outcomes regarding the viability of *A. myosuroides* seeds closely mirrored those of the germination results for this weed species. Once again, it was evident that a significant loss of seed viability occurred only after an extended duration in the rumen, particularly in the presence of the diet designed for lactating cows.

Both the germination and the viability of *A. retroflexus* were influenced only by the incubation time (P > 0.01) and less evidently by diet (P = 0.023). The germination of *A. retroflexus* seeds remained relatively high with a slight induction compared to the control during short and medium incubation times. However, a slight reduction of germination was observed at longer incubation times. As for the diets, there were variations in the germination percentage when the seeds of *A. retroflexus* were exposed to the rumen fermentation with different diets. More seeds germinated when exposed to heifers’ diet compared to those for lactating cows, but the difference was modest. Similar to the germination results, for seed viability, the only significant influencing factors were the incubation time (P< 0.001) and only marginally by the diet (P = 0.021), but there was no influence of the diet x incubation time interaction. The seed viability percentage remained quite high, with only a slight reduction observed after an extended duration in the rumen. These results align with the observations made for the germination of *A. retroflexus* seeds, suggesting a slightly higher survival rate with heifers’ diet compared to that for lactating cows.

The only factor influencing the germination and viability of *A. sterilis* was the incubation time (P< 0.001). Both seed germination and viability of *A. sterilis* were remarkably low after passage through the simulated cattle digestive system (**Figures 1**, **2**). Even at the shortest exposure time, less than 20% of the seeds germinated, and this percentage further decreases with increasing incubation time.

The results concerning the viability and germination of *C. album* after the passage through the cattle digestive system revealed significant patterns. Data from **Table 4** illustrates that incubation time, diet and their interaction had significant impact on germination (**Figure 1**). However, none of the factors included in the statistical model had significant effect on the viability of *C. album* seeds, which was maintained very high and close to 100%. The results reveal an increase in the germination rate of seeds for each time x diet combination compared to the control (**Figure 1**). Notably, the highest germination percentage was observed for seeds exposed to rumen activity for 48 hours. Additionally, it is noteworthy that in this species the diet of lactating cows induced more germination, even at shorter permanence periods in the rumen, compared to that of the heifers.

The results regarding the germination of *D. stramonium* seeds were distinctive. It was evident that compared to control both diets reduced the germination percentage of the seeds, especially after 48 hours of permanence in the rumen. However, this reduction in germination was more pronounced with heifers’ diet, considering that the germination percentage remained quite high after 12 and 24 hours. Meanwhile, for lactating cows’ diet, the reduction in germination over time was less abrupt, even though it exhibits lower values even at shorter permanence in the rumen. The results obtained concerning the seed viability of this weed species (**Figure 2**), suggest that the reduction in seed viability becomes significant only after an extended permanence in the rumen, specifically after 48 hours. Notably, after 48 hours, more seeds of this species lost their viability under the influence of the heifers’ diet compared to those of lactating cows.

The data of *E. crus-galli* present in **Table 5** show that, also for this species, there was no significant impact on the seed viability after the passage through the cattle digestive system. On the other hand, the incubation time significantly impacted the germination of this weed species. Each incubation period had a promoting effect on *E. crus-galli* seed germination compared to the control (**Figure 1**). This effect is particularly noticeable after 48 hours of incubation (P<0.001).

The seed viability and germination of the seeds of *L. multiflorum* were affected by the diet and the incubation time, but not by their interaction (**Tables 4**, **5**). In addition, as in the case of *A. sterilis*, the only viable seeds were the ones that germinated. The results demonstrate that each incubation time led to a reduction in the seed germination of *L. multiflorum* compared to the control (**Figure 1**). However, even though there were significant differences indicating a major reduction in seed germination after 48 hours of incubation, it is noteworthy that the germination percentage remained higher than 60%. In addition, seeds exposed to the lactating cows’ diet exhibited a lower germination percentage compared to those of heifers.

Ultimately, the results concerning both seed germination (**Table 4**) and seed viability (**Table 5**) showed no significant influence of any of the factors or their interactions on *S. halepense*. Meaning that the seed of this weed species remained intact during this process.

### 3.3 Cellulolytic properties of the rumen fluid and seed viability

**Figure 3:**
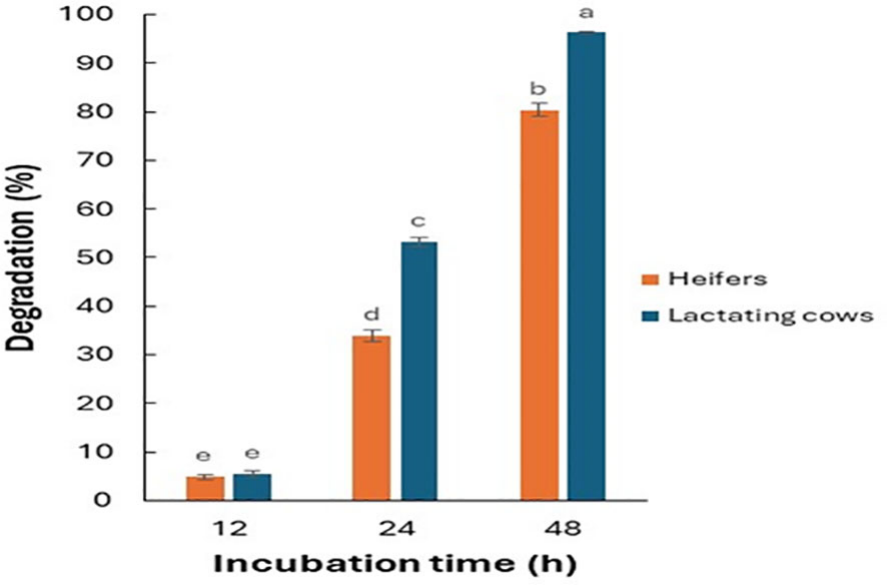
Degradation percentage of cotton threads, as estimation of rumen microbial activity, exposed to different diets and to different incubation times. The letters indicate a significant difference *p*-value< 0.05 (Nikolić et al., 2024).

**Figure 4:**
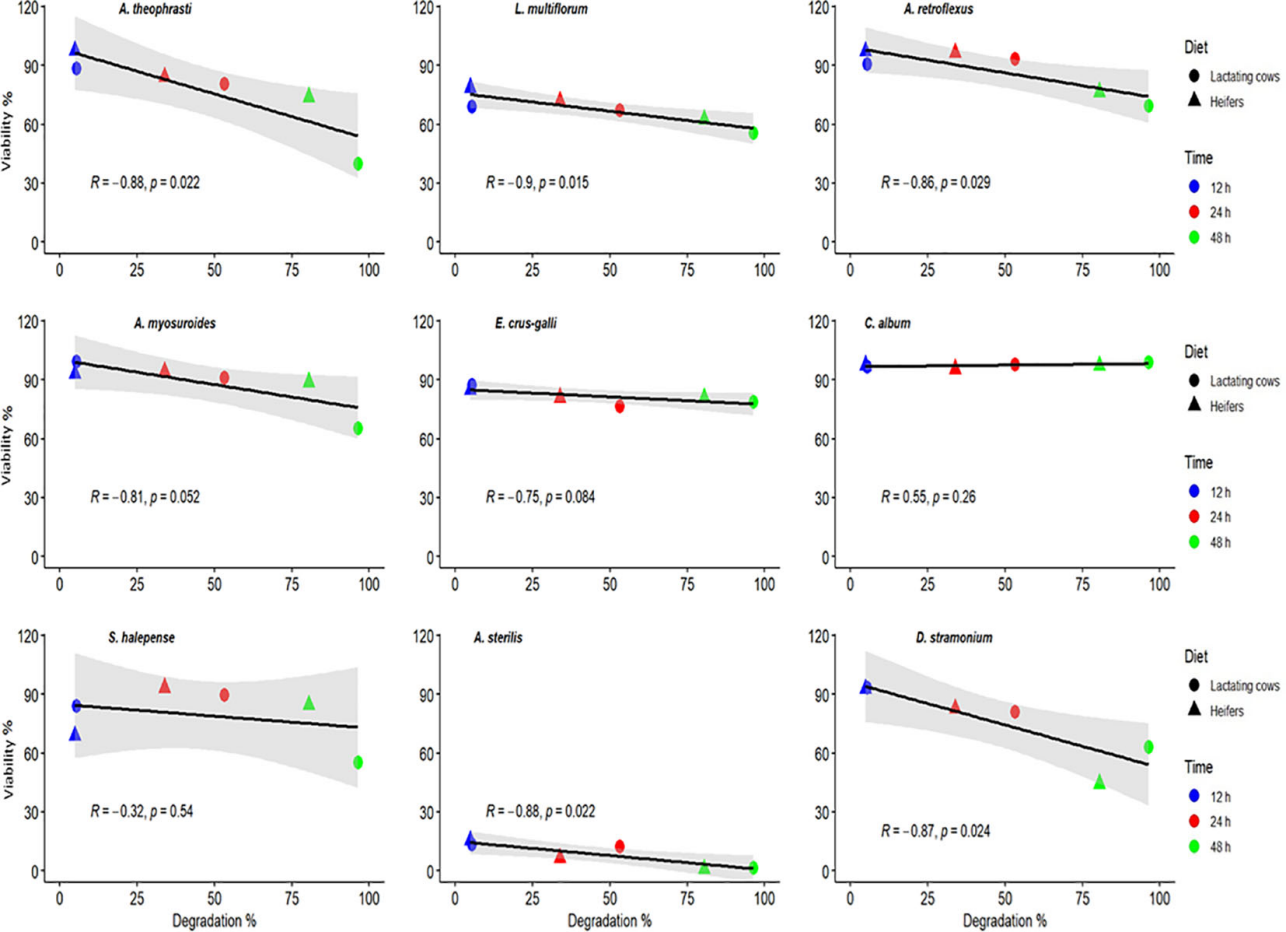
Correlation between the microbial activity measured inside the rumen at different times and estimated weed seed degradation.

Data from the Fertimetro suggested a progressive increase in cellulolytic microbial activity of the rumen fluid with increasing incubation times (**Figure 3**). At 24 and 48 h of incubation, microbial activity was greater in the diet for lactating cows compared to that of heifers. As evidenced in **Figure 4**, there were negative correlations (from -0.32 to -0.90) between the cellulolytic properties of the rumen fluid, measured from the cotton threads resistance at pulling, and the seed viability after the passage through the simulated digestive system. As expected, there was no significant correlation for the same species that also exhibited little to no changes in their viability after being exposed to rumen microbial activity (*E. crus-galli*, *C. album*, *S. halepense*). Furthermore, depending on the species and the composition of the seed coat, this measurement may also suggest a higher germination percentage for seeds in environments with elevated cellulolytic microbial activity. In such environments, the microbial activity may effectively scarify the seed coat, facilitating the initiation of the germination process. Conversely, in environments with lower microbial activity, the seed coat may remain intact, hindering germination. The Fertimetro method has proven to be indicative of seed degradation in rumen, as observed in other environments, such as soil (Nikolić et al., 2020, 2024).

## 4 Discussion

The seeds of various weed species exhibit different shapes, sizes, and compositions, including variations in cellulose, hemicellulose, and lignin content (Biswas et al., 1970). These distinctions in seed-building materials can significantly impact seed degradation, particularly when influenced by microorganisms (Davis et al., 2008; Krzyzanowski et al., 2023; Talbot and Treseder, 2012). The results of our study unveiled distinct behavioral patterns among various weed species concerning germination and seed viability following their passage through the cattle digestive system. This diversity aligns with existing research, affirming the wide range of degradation percentages exhibited by seeds of different shapes and sizes from varied plant families when subjected to the cattle digestive system (Blackshaw and Rode, 1991; Lowry, 1996; Ocumpaugh and Swakon, 1993). Both seed germination and viability are intricately dependent on a myriad of biotic and abiotic factors and their interactions (Humphries et al., 2018; Javaid et al., 2022; Nikolić et al., 2020; Ranganathan and Groot, 2023). Monitoring the effects of these factors and their interactions becomes paramount, especially within environments like the cattle rumen.

### 4.1 Chemical composition, seed germination and viability

The thickness and composition of the seed coat are usually considered crucial determinants in the degradation process, where species with larger seeds and thicker seed coats demonstrated prolonged survival under adverse conditions compared to species with smaller seeds and thinner seed coats (Dubbern de Souza and Marcos-Filho, 2001; Manonmani et al., 1996). The constituents of the seed coat, particularly lignin, cellulose, and hemicellulose, play pivotal roles in shielding the embryo from adversities, either individually or through their intricate interactions (Polo et al., 2020; Abati et al., 2022; Brzezinski et al., 2022).

The results from the current study align with these statements, with all species, except *A. sterilis*, maintaining viability throughout the trials or experiencing significant viability reduction only after 48 hours of incubation. Analysis of seed composition (**Table 3**) reveals that the seed species studied exhibit high content of cellulose, hemicellulose, lignin, or a combination of these. Notably, *A. sterilis* stands out with low content in all three substances. Given that these compounds are primarily located in the cell walls forming the seed coat, tasked with protecting the embryo (Grgas et al., 2023; Krzyzanowski et al., 2023; Polo et al., 2020; Ranganathan and Groot, 2023), their concentration likely exerts a significant influence on seed degradation.

Contrary to expectations, seed size alone exhibited minimal influence on degradation outcomes. Differently from *A. sterilis* with large seeds, *S. halepense*, *A. theophrasti*, and *D. stramonium* with equally large seeds maintained high seed viability. The latter three species exhibited this resilience due to their elevated content of cellulose, hemicellulose, and lignin compared to *A. sterilis*. In contrast, certain species such as *C. album*, *S. halepense*, and *E. crus-galli* displayed no effects on seed viability after passage through the cattle digestive system. The resistance of *C. album* can be attributed to its high lignin content (24.14%), while *S. halepense* relies on its abundant hemicellulose and cellulose. For *E. crus-galli*, whose content of these substances is medium to low, the puzzle may be unraveled by exploring different substances produced by the seeds, potentially possessing antimicrobial properties. Research by Odintsova et al. (2008) and Rogozhin et al. (2012) identified specific polypeptides named defensins in *E. crus-galli* seeds, expressing antimicrobial properties. Although our study did not delve into this investigation, the presence of these defensins may underpin the survival of *E. crus-galli* seeds and warrants further exploration.

### 4.2 Resistance of the seeds in the ruminant digestive system and manure

A significant trend emerged among species influenced by the cattle digestive system, with the inoculum from lactating cows exerting a greater impact on germination and viability compared to that from heifers. These differences are mainly due to the different chemical composition of the diets, which influences the composition and activity of the ruminal fluid. As expected, the higher starch content in the lactating cows’ diets, compared to those of heifers, stimulates microbial activity, resulting in increased volatile fatty acid concentrations and a lower ruminal fluid pH.

The digestive effects observed with the *in vitro* approach applied in the current paper is not fully representative of the *in vivo* conditions, where the fodder is continuously chewed because of rumination, and moved along the digestive lumen. However, the *in vitro* simulation of the digestive processes was well reflected by the degradation of the cotton threads, which distinguished pretty well the effects of the two inoculum and diets on the cellulolytic activity of the rumen microbes. The positive correlations found between the seed viabilities and the cellulolytic properties of the rumen fluid aligns with previous findings of Nikolić et al. (2020), who evidenced a direct relationship between increased cellulolytic microbial activity and heightened seed degradation.

Beyond their protective role, seed coats can present challenges for germination (Egley, 1989). While the seed coat acts as the primary defense for the seed embryo, germination and the subsequent emergence of a new plant necessitate partial or complete destruction of the seed coat (Mohamed-Yasseen et al., 1994; Muralidhara et al., 2016). This is particularly pertinent for species with very thick or resistant seed coats. Although complete destruction may risk embryo damage and seed death, minor damages, known as scarification, can stimulate seed germination (Paudyal et al., 2021; Jarrar et al., 2023). Germination-stimulating effects can also be obtained through microbial scarification (Cardarelli et al., 2022), this could explain the results of germination after the passage of the seeds through the digestive system.

Seeds with thicker seed coats, such as *A. theophrasti*, *C. album*, and *E. crus-galli*, exhibit germination stimulation when subjected to the ruminant digestive environment. This aligns with studies demonstrating improved germination in these species after scarification (Horowitz and Taylorson, 1985; Kinugasa et al., 2016; Paudyal et al., 2021). *A. theophrasti*’s germination percentage tends to decrease over time, with lactating cows’ diet significantly reducing it after 48 hours of microbial activity. This emphasizes the stronger cellulolytic microbial activity in lactating cows’ diet, potentially damaging seed embryos after prolonged rumen exposure. Despite this, studies indicate the presence of *A. theophrasti* seeds in cattle manure, even from dairy farms, suggesting resilience even under lactating cows’ microbial activity (Pleasant and Schlather, 1994). For *C. album*, the germination rate increases with incubation time. At low to medium incubation times, germination is higher with lactating cows’ diet, with no difference after 48 hours. The heightened microbial activity in lactating cows’ simulation likely accelerates seed coat scarification compared to heifers’ diet. As for *E. crus-galli*, germination is stimulated by rumen permanence, increasing with incubation time. This aligns with studies indicating that seed scarification induces germination in this weed species (Sung et al., 1987; Paudyal et al., 2021).

In an exception that confirms the rule, *D. stramonium* seed germination is significantly reduced compared to control, especially after prolonged rumen incubation. Brown and Bridglall (1987) found that seed scarification induces germination in *D. stramonium*, while Nikolić et al. (2022) demonstrated germination at extremely low pH values. The nature of the cattle rumen, with seeds immersed in rumen liquid, may explain this behavior, as certain species in this genus do not respond well to soaking (Reisman-Berman et al., 1989). Therefore, *D. stramonium* germination in the cattle digestive system may be more linked to the physical properties of the rumen than to chemical or microbiological factors.

Conversely, seeds with thinner seed coats, as exemplified by *A. sterilis*, and even *L. multiflorum*, experience rapid loss of germinability and viability when subjected to the digestive environment. The latter, however, retains a significantly greater proportion of germinable and viable seeds compared to *A. sterilis*. A closer look reveals that *L. multiflorum* seeds have slightly higher concentrations of fibrous compounds, coupled with smaller seed sizes, potentially contributing to their enhanced survival. Moreover, *L. multiflorum*, often used as forage, exhibits viable seeds found in cattle excrements, indicating potential adaptation to endozoochorous dispersal (Stanton et al., 2002). For seeds with thin seed coats like *A. retroflexus* and *A. myosuroides*, germinability remains high after short to medium exposure to microbial activity. Interestingly, both species exhibit higher germinability loss when subjected to the lactating cows’ diet than heifers. The higher lignin content in *A. retroflexus* seeds likely contributes to its survival. Additionally, peptides with antimicrobial properties, identified by Lipkin et al. (2005) in *A. retroflexus* seeds, could play a role in maintaining germinability. Despite lacking germination stimulation effects, *A. retroflexus’*s prolific seed production, coupled with herbicide-resistant populations, raises concerns about potential introductions and spread through cattle trade globally (McLachlan et al., 1995; Costea et al., 2004; Yanniccari et al., 2022). *A. myosuroides* seeds, possessing low lignin but higher concentrations of cellulose and hemicellulose, likely shield the embryo during low to medium exposure to microbial activity. Our findings align with Milotić and Hoffmann (2016), affirming similar effects on seed germinability post-cattle digestive system passage.

Finally, it is important to highlight some potential limitations of the current research. An important limitation might be related to the *in vitro* simulation of the animal digestion process, which cannot fully replicate the complex dynamics of digestion within the rumen, gastric, and intestinal compartments. *In vivo*, seeds move gradually through the gastrointestinal tract, following a Gaussian distribution, with some seeds excreted within a few hours and others much later. This is influenced by factors such as seed size and feed composition, where smaller seeds may travel faster and larger seeds may remain in the digestive system longer (Gardener et al., 1993; Lepková and Mašková, 2024).

On the other hand, the *in vitro* method exposes all seeds to the digestive factors for the same duration, potentially oversimplifying the digestive process. To address this limitation, we adopted different fermentation times (12, 24, and 48 hours) to simulate varying scenarios of seed fate within the rumen, aiming to closely simulate the real-life conditions.

Additionally, the conditions present in the rumen, such as microbial activity, temperature, and pH, which were replicated *in vitro* to measure germination and seed viability, may not perfectly match the variability and complexity of natural *in vivo* conditions. While these standardized measures help control experimental variables, they might not fully account for the dynamic and heterogeneous environment of the rumen during digestion and seed excretion.

Furthermore, although the results obtained are supported by the abundant literature available, it is important to clarify that the primary aim of this study was not to establish a strict correlation between the chemical composition of seeds and their degradation within the cattle rumen. While previous research highlights the importance of fibrous components—such as cellulose, hemicellulose, and lignin—in determining seed survival (Westerman and Gerowitt, 2013; Madureira et al., 2023), these factors alone are insufficient to explain the degradation process fully. This is because seed digestion is influenced by a complex interplay of multiple and specific traits, including seed size, coat thickness, degree of lignification, microfibrillar structure of fibrous compounds, and the relative proportions and anatomical distribution of hemicellulose, cellulose, and lignin (Bewley and Black, 1994; Moles and Westoby, 2004).

In addition, the presence of soluble fibers, such as pectin or beta-glucans, along with the imbibition capacity of seeds, plays a crucial role in determining their response to the digestive environment (Langenaeken et al., 2020; Choudhury et al., 2023). These traits often vary significantly between species and within species due to genetic and environmental factors. Therefore, while a correlation between specific fibrous compounds and seed germination or viability may be identified in studies focusing on a single species or closely related species (Lepková and Mašková, 2024), such correlations are unlikely to hold across a diverse set of weed species with differing seed morphologies and biochemical characteristics.

It is also worth noting that the ruminal degradation process is not purely chemical; it involves microbial activity, physical breakdown, and interactions with other feed components in the digestive matrix (Brice and Morrison, 1982; Blackshaw and Rode, 1991; Westerman and Gerowitt, 2013).

While our study does not seek to resolve this complexity, but to explore the survival of some agronomically important species, is still provides valuable insights into the general patterns and trends associated with weed seed survival in the rumen. Future research, focusing on single or similar species analyses, coupled with detailed biochemical profiling and controlled digestion trials, could elucidate the relationships between seed chemical composition and degradation outcomes.

## 5 Conclusion

This study reveals the intricate dynamics of weed seed germination and viability following passage through the cattle digestive system. Diverse behaviors were observed across weed species, emphasizing the influence of seed coat characteristics, microbial activity, and the compositional intricacies of cattle rumen fluid. The study underscores the significance of the chemical composition of seeds, particularly the content of lignin, cellulose, and hemicellulose in weed seed survival. However, it also highlights the complexity of natural systems, where exceptions exist and warrant further exploration.

Weed seed survival, and in some cases higher germination after passage through the cattle digestive system, raises concerns about the potential introduction of new weed species or resistant populations through cattle movement or trade. As noted in the introduction, grazing has historically been identified as a significant factor in the spread of weeds. Our findings reinforce this concern by demonstrating that seeds of certain weed species can survive passage through the digestive system of cattle and remain viable for extended periods. This highlights the potential for grazing cattle to ingest mature weed seeds in one location and deposit viable seeds in another via their feces, even after a delay of one or more days. The seasonal overlap between grazing periods and weed seed maturation exacerbates this risk, particularly given the species-specific variability in seed survival observed in our study.

Moreover, this dispersal mechanism is not limited to grazing systems. During the animal trade, cattle transported over both short and long distances may spread weed seeds, especially when they are fed with fodder sourced from their region of origin. This process could facilitate the introduction of weed populations into new locations. Importantly, the introduction of genetically distinct populations of the same species could promote interpopulation hybridization, potentially resulting in more aggressive and invasive weed variants. Our findings contribute valuable insights into the intricate interplay of factors affecting weed seed fate within the cattle digestive system. As weed management strategies evolve, understanding these nuanced dynamics becomes essential for mitigating potential risks associated with the spread of herbicide-resistant populations, ensuring sustainable agricultural practices.

Furthermore, this study provides a snapshot of weed seed behavior post-digestion in cattle. Additional research examining weed seeds in feed and manure is crucial to closing the cycle from field to field. Given the high adaptability potential and constant evolution of weed species, their seed behavior remains puzzling, even as we continue to assemble the pieces.

## Data Availability

The raw data supporting the conclusions of this article will be made available by the authors, without undue reservation.
