# Supplementary material for: Post digestion weed seed survival in cattle
Source: Front Plant Sci. 2025 Mar 18;16:1483774. doi: 10.3389/fpls.2025.1483774 (PMC11960749; doi:10.3389/fpls.2025.1483774)
Supplement: Supplementary file 1 [file Table1.docx]

Supplementary Table 1. Mean percentage (%) of germinated, dormient and viable weed seeds of control sampling.

| Species^1^ | Germinated | Non germinated | Dormient | Degraded | Viable |
| --- | --- | --- | --- | --- | --- |
| ABUTH | 37.25 | 62.75 | 33.5 | 29.25 | 70.75 |
| ALOMY | 94.75 | 5.25 | 0.25 | 5 | 95 |
| AMARE | 88.25 | 11.75 | 0 | 11.75 | 88.25 |
| AVEST | 79.5 | 20.5 | 0 | 20.5 | 79.5 |
| CHEAL | 53.75 | 46.25 | 10.25 | 36 | 64 |
| DATST | 63 | 37 | 9.75 | 27.25 | 72.75 |
| ECHCG | 26.5 | 73.5 | 5 | 68.5 | 31.5 |
| LOLMU | 99.5 | 0.5 | 0 | 0.5 | 99.5 |
| SORHA | 17.25 | 82.75 | 8 | 74.75 | 25.25 |

^1^ ABUTH: *Abutilon theophrasti* Medik., ALOMY: *Alopecurus myosuroides* Huds. AMARE: *Amaranthus retroflexus* L., AVEST: *Avena sterilis* L., CHEAL: *Chenopodium album* L., DATST: *Datura stramonium* L., ECHCG: *Echinochloa crus-galli* [L.] P. Beauv., LOLMU: *Lolium multiflorum* Lam., SORHA: *Sorghum halepense* L
